# Patient and Therapist Experiences of the SaeboFlex: A Pilot Study

**DOI:** 10.1155/2017/5462078

**Published:** 2017-01-09

**Authors:** Larissa Andriske, Debbie Verikios, Danielle Hitch

**Affiliations:** ^1^Barwon Health, Geelong, VIC, Australia; ^2^Deakin University, Geelong, VIC, Australia

## Abstract

**Objective:**

The aim of this pilot study was to explore the experiences of both patients and therapists of using the SaeboFlex.

**Method:**

A mixed methods approach was adopted. Patients completed a questionnaire which included the Psychosocial Impacts of Assistive Devices Scale (PIADS) (Version 3.0) and 6 qualitative questions. Therapists completed 2 qualitative questionnaires, which collected data on the regimen adopted for the SaeboFlex and reflections on their practice with this device.

**Results:**

The SaeboFlex had a mostly positive impact on both the psychosocial experience of patients and their ability to do, be, and become. Intervention regimens were broadly similar between therapists, and both patients and therapists stated that the SaeboFlex increased motivation for therapy.

**Conclusion:**

This study has added to our tentative understanding of the SaeboFlex, but more rigorous research is required to build a robust evidence base.

## 1. Introduction

The SaeboFlex is a dynamic custom fabricated wrist, hand, and finger orthosis (WHFO). As a purely mechanical spring assisted device, the SaeboFlex enables people with decreased wrist, hand, and finger function to participate more actively in both rehabilitation and daily occupations. The SaeboFlex is considered particularly suitable for patients with insufficient wrist and finger extension to participate in repetitive task oriented therapy, including those who have experienced stroke, spinal cord injury, and acquired brain injury [1].

The SaeboFlex has been on the market for several years, and a small body of evidence has now emerged about its effectiveness. A pre-post study with people experiencing chronic poststroke hemiparesis followed up participants who engaged in a total of 30 hours of training with the device. Improvements were found in shoulder and elbow movement, wrist extension, and muscle tone [2]. A further series of case studies were completed with people following severe stroke, who used the SaeboFlex for just 1 week for a total of 3 hours [3]. Small gains were found in reaching and grasping, and the subject found to make the greatest gain had a primary problem of weakness. The two conference abstracts have also reported pre-post studies with people following stroke. The first reported significant increases in grip strength and wrist extension following a 6-week program involving use for 90 minutes per day [4]. The other reported improvements in active shoulder flexion and abduction, decreased muscle tone, and improved motor recovery and grip strength [5]. This study also used an occupational therapy outcome measure focusing on meaningful activities and found that 80% of the participants achieved their personal functional goals after the 5 months of the program.

Most recently, a clinical case series study focused on the feasibility of SaeboFlex upper-limb training in acute stroke rehabilitation [6]. A sample of 8 patients with less than 3 months after stroke were recruited and participated in a 12-week individualized training program (of up to 3 × 45-minute sessions per day). Clinically significant improvements were found in the majority of participants for upper-limb function, although one participant experienced shoulder complications. The authors concluded that the SaeboFlex has the potential to improve participation and independence in activities of daily living but did not recommend further research.

There have also been two descriptive accounts of the experiences of therapists with the SaeboFlex [7, 8], which were generally very positive. However, the experience of patients in using this device has not been the subject of research to date, nor has the use of the device with people other than those who have had a stroke. The aim of this pilot study was therefore to explore the experiences of both patients and therapists of using the SaeboFlex, as a first step towards understanding how it can be successfully translated into regular practice.

This pilot study focused on specific aspects of the patient and therapist experience of the SaeboFlex, which were identified as crucial to its uptake and sustainability as a part of therapy. The research questions addressed were the following:

1. What impact did the SaeboFlex have on the psychosocial experience of patients undergoing neurological rehabilitation?
2. What perceived impact did the SaeboFlex have on the patient's ability to do, be, become, and belong?
3. How did the therapists utilize the SaeboFlex in practice?
4. What were the therapist's reflections on his/her experience of using the SaeboFlex with his/her patients?

## 2. Method

This pilot study was conducted using a mixed methods approach, with both quantitative and qualitative data collected from patients and therapists. Ethics approval was sought and provided by both the health service in which the study was based and the university which provided research support.

### 2.1. Procedure

Several different methods of data collection were used, incorporating both standardized and nonstandardized methods. The inclusion criteria for patient participants were (1) patients with a neurological condition resulting in hemiparesis and (2) patients whose treatment has included use of the SaeboFlex within the Occupational Therapy Program between January 2012 and December 2013. Patients with mental health or cognitive issues which impacted on their ability to give informed consent were excluded from the study.

At the conclusion of their program with the SaeboFlex, patients were invited to participate and, if agreeable, given a single questionnaire to complete either individually or with the help of a carer. The questionnaire began with the Psychosocial Impacts of Assistive Devices Scale (PIADS) (Version 3.0), which addressed the first research question. The PIADS has been standardized and measures quality of life outcomes around the use of assistive technology via a 26-item self-rating scale [9]. There are 3 subscales (competence, adaptability, and self-esteem), with each item rated from* −3 (maximal negative impact)* to* +3 (maximal positive impact)*. The PIADS has been shown to have good construct validity, test-retest stability, reliability and internal consistency, and acceptable concurrent validity [10–12]. Following the PIADS, patients were asked 6 open-ended questions seeking their comments on how the SaeboFlex supported their ability to do, be, become, and belong. These dimensions originated in Wilcock's [13]. Occupational Perspective of Health, although the updated definitions formulated by Hitch et al. [14] were provided on this questionnaire for the patient's reference. Single questions were devoted specifically to each dimension, while a further question asked the patient to comment on whether the SaeboFlex had affected his/her personal experience and a final question asked for any further comments. Please see the Appendix for the full list of qualitative questions posed.

All occupational therapists (known as therapists from this point on) working at the health service who had utilized the SaeboFlex were eligible to take part in this study. At the conclusion of their work with a patient using the SaeboFlex, therapists were invited to participate and, if agreeable, were asked to provide qualitative data regarding their experience of the device. The questionnaires included space to record the patients' medical and social history, the regimen of use adopted for the SaeboFlex, the patients functional goals reflections on clinical reasoning, opinion on usefulness in practice, and positive and negative experiences. While all this data has contributed to quality assurance activities at the service, only data related to the regimen adopted, clinician perceptions of utility, and experience of the device are reported here. These data were collected separately to keep the reflective data confidential, as occupational therapy students contributed to the extraction of medical and social history data from the patients file onto the first form. Analysis only commenced once all forms of data had been collected.

### 2.2. Data Analysis

Given the pilot nature of this study, all quantitative data from patients were analyzed descriptively by frequencies, means, and standard deviations. All qualitative data were transferred to a separate Microsoft Word document. Responses specifically related to the dimensions of occupation (doing, being, becoming, and belonging) were coded in relation to these dimensions; for example, responses which alluded to the patients aspirations were included in data related to becoming. These data were then analyzed thematically, and direct quotes have been extracted to illustrate each theme in the words of the patients.

Qualitative data from therapists around the utilization of the SaeboFlex were analyzed quantitatively using the first 7 sections of the TIDieR template for intervention description [15]. This template aims to encourage the complete description of published interventions, as a means to improving replicability in the longer term. The final 3 sections of this template (Tailoring, Modifications, and How Well) relate to highly variable factors, which cannot be adequately reported in a pilot study. Content analysis was used to quantify the responses required, with regimen features applied to three or more patients reported. Qualitative data from therapists related to the use of the device with their patients were analyzed thematically, with themes allowed to emerge from the data.

## 3. Results

### 3.1. Sample

Eleven patient participants were recruited and completed both sections of the questionnaire. Seven participants (64%) were male, with around half of them (54%) being in their 60s. While the mean age was 60.2 (SD = 17.89), the youngest participant was in their mid-20s and the oldest participant was in their early 90s. While we had hoped to recruit participants with a range of diagnoses, all participants had experienced a stroke (with one also diagnosed with a brain tumor).

Five therapist participants were recruited, all of whom completed reflective responses for their work with clients (resulting in 11 records). Eight SaeboFlex intervention regimens were submitted for analysis. All patients were also using other treatment modalities along with the Saebo, including e-stim, wrist bracing, splinting, and/or casting, although the time after injury for each of the participants when using the SaeboFlex was variable. All were presenting with moderate to severe spasticity in their wrist and fingers, and none of them had any functional grasp or release movements in their upper limbs. All of these therapists were employed by a large regional health service and worked across both subacute and community services.

### 3.2. Patients

#### 3.2.1. Psychosocial Experience

**Figure 1:**
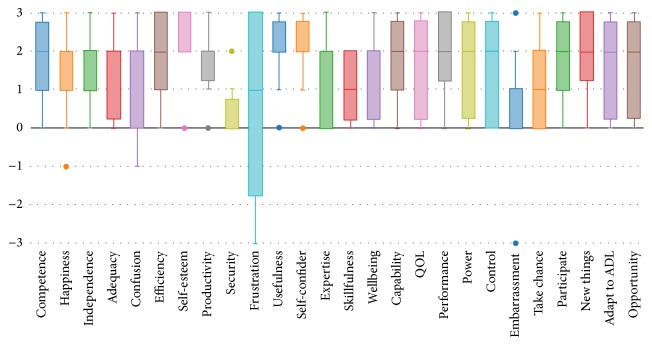
Box-and-whisker plot of PIADS ratings for the SaeboFlex from all participants.

As displayed in Figure 1, the patients experienced mostly positive psychosocial experiences as a result of using the SaeboFlex. The box and whisker chart displays the interquartile ranges (boxes), maximums and minimums (whiskers), and outliers (dots) from all responses. The only item which elicited more than one negative response was “frustration,” and qualitative comments associated with this item identified the customized fitting process as the source of this response.

#### 3.2.2. Perceptions of Doing, Being, Becoming, and Belonging

*(1) Doing*. The majority (*n* = 10) of the patients noted they were able to do more when using the SaeboFlex, citing improvements in range of movement, strength, grip, and control;* “the more practice I had the more my ability to progress towards independence.”* However, the amount of progress varied amongst the patients, some of whom were in the acute phase and some were up to several years after stroke;* “Still not able to use my hand functionally, but have noticed increased strength, increased control of movement.”*

*(2) Being*. Individual confidence and motivation were the 2 main aspects of being identified by the patients (*n* = 8), with the SaeboFlex found to be supportive of both. Successful doing was a key factor in the building of confidence, which was also generalized by the patients to other activities of daily living;* “it definitely build my confidence to attempt to try other things because I knew that my brain was remembering the movement.”* While some patients (*n* = 4) reported fluctuating motivation through their time using the SaeboFlex, the majority acknowledged that its use had contributed to maintaining their engagement in the rehabilitation process;* “It gave me a positive mind set and was the start of the hardest thing I have done in my life.”*

*(3) Becoming*. Hope was a very strong theme in the data related to the occupational dimension of becoming (*n* = 8), with patients consistently highlighting a lack of self-belief in their abilities at the beginning of their use of the SaeboFlex; “*it helped me use my hand even though I thought I never would.”* The realization that a return to function was possible was very powerful for some of the patients;* “My hand moved, I couldn't believe it! I finally could see a shining light and I was going to get some movement back in my arm and hand with hard work and encouragement.”* This sense of hope also encouraged further aspiration, as patients looked forward to achieving future goals as a result of their use of the SaeboFlex;* “I'm hoping to get to the stage where I can open jars that are too hard for my wife.”*

*(4) Belonging*. The majority of patients (*n* = 9) stated that the SaeboFlex did not impact on their sense of belonging to any appreciable degree. Their rehabilitation using this device was perceived as a very personal and individual experience, although two patients could see a link between their gains in doing and participation in social activities;* “I feel using the SaeboFlex will help me be more independent and less reliant on my family and friends.”*

### 3.3. Occupational Therapists

#### 3.3.1. Use of SaeboFlex in Practice

The consistent rationales cited for using the SaeboFlex were to (1) improve upper-limb functional performance, (2) improve active range of movement, (3) improve grip strength, and (4) provide a means for ongoing home based rehabilitation. The main material used was the SaeboFlex orthosis in combination with specific sized balls for use in rehabilitation exercises. The therapist was the only person providing the intervention, and the SaeboFlex was used individually in all cases. In this study, the SaeboFlex was used within both the clinical setting and the home environment. For home programs, equipment was provided on loan to the patients from the health service, with some patients going on to seek funding to purchase the SaeboFlex for ongoing use. As shown in Table 1, there was some variation in regard to when and how much the SaeboFlex was used. The shortest duration of SaeboFlex use was one week, although many regimens lasted 2-3 months. Sessions were either 20 or 45 minutes long, and all were performed at least once on a daily basis. Repetitions (i.e., each instance of grasp and release) tended to increase over time ranging from 20 to 150 per session.

#### 3.3.2. Use of SaeboFlex in Practice

Themes elicited from data related to therapist experiences in using this device were benefits and barriers. The main benefit of using the SaeboFlex highlighted was the supportive impact it had on patient motivation;* “A few short sessions with SaeboFlex was enough to ‘light a fire' to get him (to) increase the use of his left upper limb.”* This was consistently identified by all therapists and appeared to be related to the rapid improvements observed in patient function:* “Quick results, quick translation into increased hand function.”* This also elicited positive emotional responses, with several therapists describing the excitement they, the patients, and their families felt in response to these improvements.

The SaeboFlex also enabled therapists and patients to focus on the upper limb more intensely,* “Provided an opportunity to attend to left upper limb for extended periods.”* However, unexpected and more generalized benefits of this intervention were also reported around improved standing tolerance, better transition to other treatment modalities, decreased neglect, and better mental state;* “It was immediately motivating and improved her mood…she was very determined during therapy and she persisted in using the SaeboFlex.”* The flexibility possible in treatment activities was also highlighted as a benefit by therapists, who stated they enjoyed the challenge of finding new things for their patients to do;* “Ability to increase use of SaeboFlex is inspiring for clients who want a challenge.”*

The main barrier experienced in using the SaeboFlex with patients was the need to have assistance in donning and doffing the orthosis. While some patients were able to do this independently, others were reliant on carers which impacted on their ability to use it at home;* “He was alone during the day and couldn't complete by himself.”* The intensity of the regimens was also seen as a potential barrier if the patient found the repetitions boring or it overtook his/her occupational balance;* “As a therapy have been mindful not to overload patient with too much to do at home so that he doesn't have time to do other things and gets burnt out or disinterested.”* Finally, a few therapists noted that the device is fairly expensive and may be beyond the means of many clients to use on an ongoing basis.

## 4. Discussion

This pilot study has achieved its aim of exploring the experiences of both patients and therapists of using the SaeboFlex, by answering all four of the research questions posed. The SaeboFlex was found to have a mostly positive impact on the psychosocial experience of patients undergoing neurological rehabilitation, although two patients expressed frustration around the fitting procedures required. Patients' perceptions of a device impact on their quality of life and wellbeing is crucial to their engagement with this form of therapy [12], so this could indicate potential for sustained use of the SaeboFlex during rehabilitation. The generally positive perception of the SaeboFlex was also found in qualitative comments from patients, who reported they were able to achieve greater function with this device and experience increased confidence and motivation. The strong theme of hope was striking in this study and supports other findings that mastery is a hope promoting factor for people recovering from stroke [16]. The lack of relationship between SaeboFlex use and belonging may be related to individual patient goals, with several patients noting that they perceive their recovery from an individual perspective.

The intervention regimens used by therapists in this study were broadly similar, with variations likely to be the results of programs being individualized to meet personal needs. Similar to Stuck et al. [6], the device was not used to the maximum recommended level (2 × 45 minutes daily) but many benefits were still observed and reported. Therapists were also able to identify barriers to using the SaeboFlex with their patients, which indicates an appropriately critical approach to its translation to practice. There were synergies between the patient and therapist experience around motivation and the motivating impact of visible results, although none of the patients specifically mentioned difficulties around donning and doffing the device.

The value of gathering perspectives from multiple stakeholders includes the possibility of highlighting different aspects of the use of the SaeboFlex, and the collection of both patient and therapist qualitative data was a strength of this pilot study. However, there were also several limitations which limit the generalizability of these findings. The sample included was quite small (for both patients and therapists), and all were recruited from a single geographical area. Without a control group, the magnitude of the improvements and benefits reported cannot be objectively measured. The variation in regimens prescribed is also a confounding factor and mitigates against the formulation of robust clinical practice guidelines at this point.

## 5. Conclusion

This pilot study has found that both patients and therapists perceive many benefits and some barriers to using the SaeboFlex as part of neurological upper-limb rehabilitation. While this study has added to our tentative understanding around how this device could successfully translate into regular practice, far more research is required to build a robust evidence base. Future studies should include data from both patients and therapists to gain a comprehensive understanding of how best to utilize the SaeboFlex and would benefit from being conducted with larger samples which include people who have experienced a range of diagnoses.

## Figures and Tables

**Table 1:** SaeboFlex regimens.

Regimen	Overall length of use	Session time and frequency	Repetitions
1	2 months	45 mins, 1-2 daily	20–40
2	3 months	45 mins, 2-3 daily	45–100
3	1 week	Daily	Not recorded
4	4 months	20 mins, 1-2 daily	50–150
5	3 months	45 mins, 1-2 daily	50–100
6	3 months	20 mins, 2 daily	Not recorded
7	1.5 months	45 mins, 1-2 daily	80–120
8	1.5 months	45 mins, not recorded	Not recorded

## Appendix

Qualitative Questions for Patients

- Do you have any further comments you would like to make on any of these ways in which the SaeboFlex has affected you?
- Do you think the SaeboFlex helped you to do the things you wanted to do? If yes, how?
- Do you think the SaeboFlex helped you to be more skilled or build your capacities? If yes, how?
- Do you think the SaeboFlex changed your hopes and aspirations – you view of what you might become? If so, how?
- Do you think using the SaeboFlex helped you to belong – to participate in relationships with others and in your community? If so, how?
- Do you have any other comments you'd like to make about the SaeboFlex, or the way it was used in your rehabilitation?
